# Temporal Population Genetics of Time Travelling Insects: A Long Term Study in a Seed-Specialized Wasp

**DOI:** 10.1371/journal.pone.0070818

**Published:** 2013-08-02

**Authors:** Marie Suez, Cindy Gidoin, François Lefèvre, Jean-Noël Candau, Alain Chalon, Thomas Boivin

**Affiliations:** 1 INRA, UR 629 Recherches Forestières Méditerranéennes, Avignon, France; 2 Natural Resources Canada, Canadian Forest Service, Great Lakes Forestry Centre, Sault Ste Marie, Ontario, Canada; University Copenhagen, Denmark

## Abstract

Many animal species experiencing spatial or interannual fluctuations of their environment are capable of prolonged diapause, a kind of dormancy that extends over more than one year. Such a prolonged diapause is commonly perceived as a temporal demographic refuge in stochastic environments, but empirical evidence is still lacking of its consequences on temporal population genetic structures. In this long-term study, we investigated how a particular pattern of prolonged diapause may influence the temporal population genetics of the invasive seed-specialized wasp *Megastigmus schimitscheki* (Hymenoptera: Torymidae) in southeastern France. We characterized the diapause strategy of *M. schimitscheki* using records of emergence from diapause in 97 larval cohorts, and we conducted a temporal population genetic study on a natural invasive wasp population sampled during ten consecutive years (1999–2008) using polymorphic microsatellite markers. We found that *M. schimitscheki* can undergo a prolonged diapause of up to five years and displays two main adult emergence peaks after two and four years of diapause. Such a bimodal and atypical pattern did not disrupt temporal gene flow between cohorts produced in even and in odd years during the period of the study. Unexpectedly, we found that this wasp population consisted of two distinct genetic sub-populations that strongly diverged in their diapause strategies, with very few admixed individuals. One of the sub-populations displayed both short and prolonged diapause (2 and 4 years respectively) in equal proportions, whereas the other sub-population displayed mainly short diapause. This study provided empirical evidence that prolonged diapause phenotypes can substantially contribute to reproduction and impact temporal genetic structures. Prolonged diapause is likely to act as both demographic and genetic refuges for insect populations living in fluctuating environments.

## Introduction

In species living in seasonal and stochastic environments, life cycle traits are strongly selected to respond to the spatial and temporal heterogeneity of abiotic and/or biotic environmental factors [1], [2]. Diapause, a kind of dormancy shown to play a key role in the evolution of life histories of animal species, has been described as an integrated response to predictable environmental fluctuations, allowing survival during portions of the year that are inappropriate for growth and reproduction [1], [3], [4]. However, when conditions are less predictable and species experience severe spatial or interannual fluctuations of their environment diapause may extend over more than one year [5]–[7]. Such a prolonged diapause is viewed as a temporal dispersal strategy [6], [8]. Although expanding diapause is associated with metabolic, survival and reproductive costs [9], it is generally thought to generate a population buffer against environmental constraints acting against non-diapausing stages [10]–[12]. In some cases, prolonged diapause may be concentrated on a single extra year of emergence [13]. But in most cases environmental conditions are less predictable and emergences of a cohort are spread over several years, with a higher proportion of individuals emerging after the minimum diapause duration (obligatory diapause) and decreasing proportions of individuals emerging over the following years [2], [6], [14].

Variability in diapause duration and its evolutionary consequences have been documented in various model systems such as insects [6], [9], [14]–[16] and crustaceans [10], [17]. Such variation in this trait has been mainly attributed to diversified bet-hedging [15], [16], [18], a risk-spreading strategy maximising the mean geometric fitness by minimising the fitness variance at the cost of lower arithmetic mean fitness [3], [4], [18]. The role of prolonged diapause as a demographic refuge in fluctuating environments has been demonstrated empirically and theoretically when the variability of diapause duration decreases population extinction risks due to unpredictable catastrophic events such as sudden limitation in food resource, enhanced predation, or pathogenic risks [12], [15], [19]. Indeed, prolonged diapause has been shown to increase the mean population growth rate in a stochastic environment, which may even facilitate the spreading phase of invasive populations when stochasticity is high [20]. Prolonged diapause may also contribute to the stability and persistence of coupled host-parasitoid interactions [11].

From a genetic standpoint, it is generally assumed that prolonged diapause may help promote the maintenance of genetic diversity, as late-emerging individuals may not experience demographic or selection events similar to early-emerging ones [17]. Studies on seed banks suggest that prolonged seed dormancy might lead to temporal substructuring of genetic diversity through a temporal Wahlund effect, where higher homozygosity is expected in the dormant stage relative to later life stages [21]. However, such an assertion remained controversial as repeated emergences and interbreeding of individuals produced in different years is likely to lead to temporally well mixed dormant stages [22]. Using joint demographic and population genetic models, Vitalis et al. [23] found that a temporal Wahlund effect due to dormancy is negligible except in very small populations. To our knowledge, there is still a lack of empirical studies specifically assessing the role of prolonged diapause in temporal gene flow within natural animal populations. The goal of the present study was to address this issue using an invasive seed-specialized wasp, *Megastigmus schimitscheki* Novitzky (Hymenoptera: Torymidae), which faces interannual fluctuations in its food resource and displays a particularly atypical pattern of prolonged diapause.

Cones and seeds of conifers are exploited by approximately 400 species of phytophagous insects worldwide [24]. Among these, spermatophagous species of the *Megastigmus* genus (Hymenoptera; Chalcidoidea; Torymidae) exhibit an extended diapause, which can be interpreted as a response to the dramatic annual variation in seed production characterizing most of their conifer host species [24], [25]. Indeed, in many conifer species, large seed crops are more or less periodically and regionally synchronized, a phenomenon referred to as masting [26], [27]. *M. schimitscheki* is an obligate predator of true cedar (*Cedrus* spp.) seeds. It was introduced in southeastern France in the early 90s in cedar seeds imported from Cyprus [28]. Its native range is the Eastern Mediterranean region, where it develops exclusively on *C. libani* Barrel in the Near East (Turkey, Syria and Lebanon) and *C. brevifolia* Henry in Cyprus [29]. Although those two cedar species were introduced to many Mediterranean countries as ornamental trees, invasive populations of *M. schimitscheki* have been observed to date solely in southeastern France. *M. schimitscheki* progressively invaded most of the planted *C. atlantica* stands in France, even showing a competitive advantage over its direct and closely related resident competitor *M. pinsapinis* (Hoffmeyer) [30], [31]. *C. atlantica* is considered a masting tree species [32], [33] and is thus thought to impose drastic and quite unpredictable variations in resource supply on its seed-specialized predators [29], [30]. As an univoltine species, *M. schimitscheki* produces one larval cohort per year. At year n, adult wasps emerge between April and June from seeds released by mature cones during the fall of year n–1. Females lay their eggs directly inside cedar ovules by inserting their ovipositor through the young cone scales, the larval instars develop within the seed by consuming the female gametophyte and the seed embryo entirely. At the end of summer, mature larvae enter diapause and first adult emergences occur only during the spring of year n+2 due to an 18-month cone maturation period, but a fraction of the larvae can extend their diapause and emerge at years n+3, n+4 or n+5 [29]. In this paper, wasps emerging 2 years after egg-laying were characterized as individuals with a short diapause phenotype (SD), while wasps emerging three to five years after egg-laying were considered to exhibit a prolonged diapause phenotype (PD).

In the present long term study, we aimed at: (i) characterizing the diapause strategy of the introduced populations of *M. schimitscheki* by estimating propensities to SD and PD within larval cohorts sampled throughout its current French distribution; (ii) assessing the temporal genetic structure of one of these natural wasp populations and testing whether or not prolonged diapause can lead to its temporal genetic substructuring; and (iii) assessing how PD phenotypes could contribute to the genetic pool of this population. Using eight polymorphic microsatellite markers, a temporal population genetic approach was developed on the oldest natural invasive population of *M. schimitscheki* in its introduced area, which has been sampled during 10 consecutive years and for which the diapause phenotype (SD or PD) of emerging individuals was identified. We showed that *M. schimitscheki* consistently displays an atypical prolonged diapause strategy that may substantially influence the temporal genetic structure of its populations.

## Materials and Methods

### Wasp Sampling and Diapause Strategy

**Figure 1:**
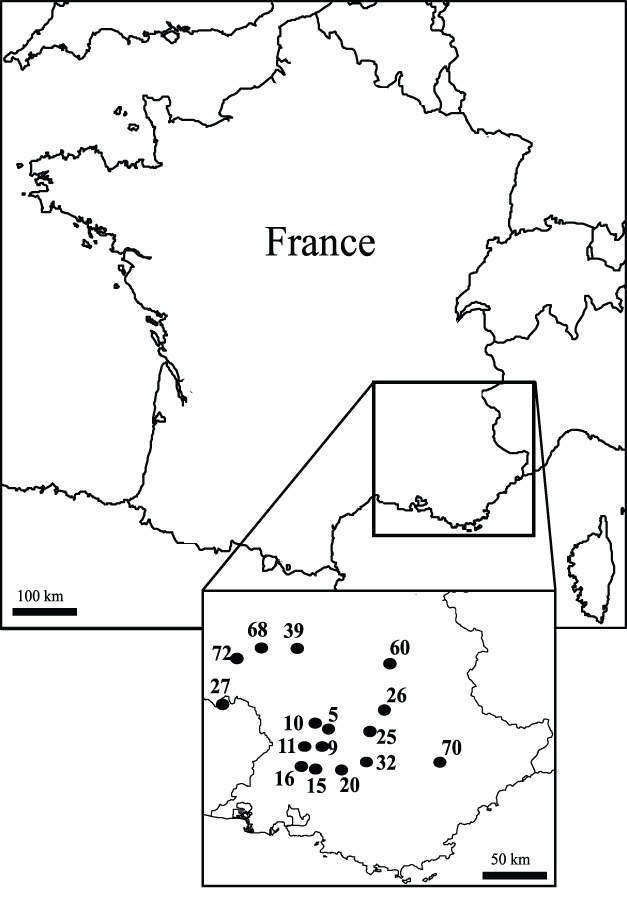
Sample locations of the 16 *M. schimitscheki* populations used in this study. Informations associated with the site codes below are provided in Table 1.

Cohorts of *Megastigmus sp.* were sampled as diapausing larvae within *C. atlantica* seeds. A cohort was defined as all the individuals that were produced the same year because *Megastigmus* species are univoltine [34]. Sixteen cedar stands were sampled throughout southeastern France from 1999 to 2008 (Figure 1). In each site, three to five cedar cones were randomly collected at 2m above the ground from 10 randomly selected cedar trees in the autumn before seasonal natural cone disarticulation and seed dispersal. Collecting cones before disarticulation ensured that the larvae found inside were produced the year before (Table 1). None of these study sites were privately owned. In France, cedar cone collection for experimental purposes does not require any specific permission in nationally owned stands or in protected areas. This study was however formerly approved by the French Ministry of Agriculture, Food and Forests (MAAP) as a contribution to the sanitary characterization of French forest reproductive material. None of the field surveys in the present study involved endangered or protected species.

**Table 1:** Sample information including the sampled localities, site codes and coordinates for a total of 97 cohorts of *M. schimitscheki* in southern France.

Site	Site code	Latitude (N)	Longitude (E)	Altitude (m)	Year of cohortproduction	N (♂–♀)
Ardènes	32	43°53′32′′	5°43′45′′	495	2001; 2006; 2007	1062 (625–437)
Barjac	27	43°47′08′′	5°07′53′′	238	2002; 2003; 2005–2007	915 (487–428)
Castellane	70	43°51′39′′	6°31′23′′	1005	2005–2007	202 (102–100)
Collet de Roland	10	44°07′45′′	5°15′36′′	1085	1999–2006	2746 (1416–1330)
Forêt de Barres	68	44°36′35′′	4°30′18′′	470	2004; 2005	123 (56–67)
Gap	60	44°32′49′′	6°00′40′′	990	2003; 2005–2007	363 (170–193)
Grand Luberon	20	43°49′61′′	5°26′32′′	1100	2000–2005	1096 (569–527)
Luberon	15	43°47′79′′	5°14′48′′	670	2000–2007	5845 (2821–3024)
Lure	25	44°0408	5°4723	960	2000–2007	4063 (2450–1613)
Mirabel	72	44]36′35′′	4°30′18′′	590	2005–2007	709 (417–292)
Oppède	16	43°49′68′′	5°09′39′′	202	1999–2005	1972 (917–1055)
Saou	39	44°39′21′′	5°06′55′′	475	2003–2007	4290 (2273–2017)
Sisteron	26	44°14′13′′	5°55′10′′	500	2000–2007	2766 (1581–1185)
St Lambert	9	44°00′06′′	5°17′30′′	789	1999–2007	4915 (2462–2453)
Mont Ventoux	5	44°08′29′′	5°23′10′′	783	1999–2008	4469 (2354–2115)
Venasque	11	43°59′7′′	5°11′57′′	320	1999–2007	6708 (3068–3640)

Cohorts were collected the year after their production due to the duration of cone maturation of their host. One cohort corresponded to a sample collected at a given site in a given year. N: total number of individuals of each sex that emerged during the five years following their production.

Collected cones were further individually disarticulated in the laboratory for manual seed extraction. Once extracted from cones, infested seeds were identified in each sample (site and year of collection) using numerical X-ray radiography (Faxitron®, 15–20 kV, 0.3–3 mA), then stored until adult emergence in rearing boxes outdoor in the Luberon cedar forest (site 15, Table 1). Seeds of *C. atlantica* can be parasitized by the 2 closely related species *M. schimitscheki* and *M. pinsapinis* [35], which are unidentifiable at the larval stage. Consequently, identification as to species was performed in the spring, at adult emergence, using morphological characters. Adults were sexed and stored in 100% ethanol at −20°C.

Adult emergences were recorded over 6 consecutive years in each of the 97 larval cohorts of *M. schimitscheki* (Table 1). Because mortality rates during diapause could not be estimated, SD propensity was defined as the proportion of adults emerging two years after their production (i.e., two-year old individuals) among all emerged individuals of a cohort. Similarly, PD propensity was defined as the proportion of adults emerging three, four and five years after their production (i.e., three-, four- and five-year old individuals) among all emerged individuals of a cohort. In the absence of PD, all the adults observed in a population at year n would belong to the same cohort produced at n–2; with PD, the adults observed at year n may belong to several cohorts n–2, n–3, etc. An additional survey of cohorts was conducted during the sixth year that followed cohort production to ensure that prolonged diapause did not exceed the five expected years. We used a linear mixed model including both site and year of cohort collection as random effects to describe the diapause pattern of *M. schimitscheki*. The predicted variable was the arcsine square root transformation of proportions of emerged adults. Both the duration of diapause (from 2 to 5 years) and the sex of adults were considered as fixed effects. Using the R-3.0.0 program [36], a likelihood ratio test and an analysis of variance (ANOVA) were performed to test random and fixed effects, respectively. To assess whether different *Megastigmus* species exploiting the same host display similar diapause strategies, adult emergences were similarly recorded for all *M. pinsapinis* individuals that emerged from the same seed lots as *M. schimitscheki*. In *M. pinsapinis* populations, thelytokous parthenogenesis generates strongly biased sex-ratios in favour of females: less than one out of 4000 sampled individuals is a male in this species [30], [34]. We thus observed only female emergences for this species. For *M. schimitscheki* only, a fraction of the emerging individuals were further genotyped.

### DNA Extraction and Microsatellite Genotyping

Temporal gene flows among cohorts of *M. schimitscheki* were estimated in the Mont Ventoux population (Table 1). The population at this site is thought to be one of the oldest in France as it is the region where the wasp was first located in the early 90s [28], [29]. Ten consecutive cohorts (1999–2008) were sampled and a total of 413 females were genotyped at 9 microsatellite loci [37], [38] (Table S1). Because wasps are haplodiploid, only diploid (i.e., female) genotypes were used to assess concordance with other recent population genetic analyses on this species [28] or on other species of this insect genus [39]. We genotyped only two- and four-year old emerged females due to extremely low emergence rates in three- and five-year old ones in the Mont Ventoux population. The numbers of SD and PD females used in each cohort for microsatellite genotyping are presented in Table 2.

**Table 2:** Propensity to prolonged diapause and indices of population genetics of ten successive cohorts (1999–20008) in a natural invasive population of *M. schimitscheki* collected at Mont Ventoux, France.

Cohort	Number of emerged males and females	% SD	% 4-year PD	N	Na	He	Ho	AR	PAR	St (P)
1999	360	55.3	36.7	32	3.00	0.55	0.60	2.74	0.00	**0.008**
2000	79	70.9	29.1	5	2.56	0.45	0.60	2.56	0.00	-
2001	140	69.3	16.4	23	2.89	0.55	0.57	2.64	0.00	**0.007**
2002	108	76.9	23.1	29	3.11	0.57	0.60	2.74	0.01	0.067
2003	556	94.6	5.0	50	3.33	0.60	0.64	2.85	0.03	0.062
2004	770	88.7	10.4	88	3.56	0.60	0.59	2.85	0.06	0.055
2005	1804	64.7	28.9	50	3.22	0.59	0.59	2.8	0.02	**0.009**
2006	289	98.6	1.4	50	3.33	0.57	0.55	2.79	0.02	0.067
2007	363	38.6	61.4	50	3.56	0.60	0.64	2.86	0.04	0.235
2008	164	18.9	79.3	36	2.89	0.58	0.69	2.77	0.07	0.067
Total	4633			413						
Mean		67.6	29.2		3.145	0.566	0.607	2.76		

SD: individuals (males and females) emerging after the obligatory 2-year diapause; 4-year PD: individuals (males and females) emerging after a 4-year prolonged diapause; N: number of genotyped females in the cohort; Na: average number of alleles; He: expected heterozygosity; Ho: observed heterozygosity; AR: allelic richness (minimum sample size of 5); PAR: private allelic richness estimated after a rarefaction procedure; St (P): probabilities associated with the rejection of the mutation–drift equilibrium using a sign test at the 0.05 threshold (probabilities lower than 0.05 are in bold).

Total genomic DNA was extracted from the entire body of each insect in individual 200 µL tubes, each containing a solution of 10% chelex resin 100 and 6 µL of 10 mg/ml proteanase K [40]. A steel ball was added to each tube and the insects were macerated in the tubes using a Qiagen TissueLyser, run twice for 10 seconds at 20 hz. The tubes with the macerated insects were then incubated at 56°C for 2 hours. After incubation the tubes were heated twice to 100°C for 15 minutes to stop the enzymatic reaction and then centrifuged at 4000 rpm to pellet the resin from the chelex resin 100. 50 µL of the supernatant containing the DNA were removed. The tubes were heated to 100°C for thirty minutes in a thermocycler, centrifuged and diluted in 30 µL H^2^0 for the PCR reactions. PCRs were performed for nine microsatellite markers distributed into two multiplex with the Qiagen® multiplex PCR kit as follows: 2 µL diluted DNA, 1 µL Q-solution(5x), 1.8 µL RNAse-free water, 5 µL QIAGEN Multiplex PCR Master mix 2x (6 mM MgCl_2_, HotStarTaq® DNA polymerase, dNTP mix), and 0.02 µL of each primer forward and reverse of 2 µM. The PCR program was 95°C 15min, 94°C 30s, 57°C 90s, 72°C 90s for 30 cycles, final elongation at 72°C for 10min and 4°C for 1 min to stop Taq polymerase in an Eppendorf thermocycler.

Electrophoresis of PCR products was performed on an ABI 3730XL sequencer as follows: 2 µL of PCR product for each individual was diluted (3 µL PCR product +50 µL H_2_O), combined with 8 µL of GeneScan500 Size Standard (10 µL GS500 (−250Liz) +900 µL Formamide), denatured at 95°C for 3 minutes and then injected into the sequencer. The microsatellite profiles were analysed in GeneMapper® software version 4.1.

File format conversions were all performed using PGDspider 2.0.1.9 [41]. FSTAT 2.9.3.2 [42] was used for calculations of allelic richness (AR) and frequencies and for the computation of mean observed and expected heterozygosities (Ho and He, respectively) over all loci for each cohort. To calculate private allele richness (PAR), we used the rarefaction procedure in HP-RARE [43], which compensates for the increase in likelihood of sampling rare alleles as sample size increases. Null allele (NA) frequencies were estimated for each locus in each cohort using GENEPOP 4.1 [44] according to the Expectation Maximization algorithm of Dempster et al. [45]. Hardy-Weinberg equilibrium (HWE) was tested using ARLEQUIN 3.5 [46] for each locus and cohort using 1000 permutation steps and 100 000 steps in the Markov chain. Linkage-disequilibrium (LD) was also tested within each cohort for all pairs of loci with 10 000 permutations using ARLEQUIN. Sequential Bonferroni corrections [47] for multiple comparisons were applied for both tests to obtain nominal significance level of 5%.

### Population Bottlenecks

As a masting tree species, *C. atlantica* displays drastic interannual variations in seed production [32], [33]. Although we did not have any data on the fructification dynamics of this tree species at Mont Ventoux during the period of the study, we tested whether the *M. schimitscheki* population there had experienced recent reductions in size possibly due to a recurrent shortage of food supply. For this purpose, bottleneck tests were performed using BOTTLENECK 1.2.02 [48] on all wasp cohorts (1999–2008), with the exception of the 2000 cohort, which was excluded from analysis due to low sample size (N = 5). The theoretical prediction is that a recent population bottleneck generates a faster reduction in allelic diversity compared to heterozygosity due to the rapid loss of rare alleles, which in turn generates an excess of expected heterozygosity in the post-bottleneck population [49]. Thus, a population bottleneck occurring in the adult population of year n–2 would be detected in the cohort of year n. In BOTTLENECK, we estimated the deviation of gene diversity averaged over loci from mutation-drift equilibrium by applying the Two-Phase (TPM) and the Stepwise Mutation (SMM) models, and using the sign test as we analysed less than ten microsatellite loci [49]. We also investigated the distribution of the allele frequencies using the mode shift test implemented in BOTTLENECK. A bottleneck in a cohort was considered significant when the three tests proved significant (sign tests under TPM and SSM, and the mode shift test).

### Temporal Population Genetic Structure

The pattern of adult wasp emergence was found to be bimodal (see Results) and suggested that >95% of the individuals of a cohort produced at a year n will emerge at year n+2 and n+4, while those issued from a cohort produced at year n+1 will mainly emerge at n+3 and n+5. Consequently, we tested if such a bimodality in adult emergence could result in a disruption of gene flow between cohorts that were produced in even years (2000, 2002, 2004, 2006 and 2008) and those produced in odd years (1999, 2001, 2003, 2005 and 2007) at the Mont Ventoux site. The temporal genetic substructuring of this population was thus tested using Wright’s *F*-statistics [50] for genetic differentiation between pooled genetic data from ‘even’ and ‘odd’ cohorts (Fst), which significance was tested using 10 000 permutation tests as implemented in GENETIX 4.05.2 [51]. The temporal genetic structure at Mont Ventoux was also analyzed using pairwise Fst estimated between all cohorts, with the exception of the 2000 one due to insufficient sample size (N = 5).

One goal of this study was to clarify the role of PD phenotypes in the temporal gene flow at Mont Ventoux. When possible in our samples, SD and PD phenotypes were distinguished among all the genotyped cohorts to define the following 12 groups of individuals: 1999-SD, 2000-PD, 2001-SD, 2002-SD, 2002-PD, 2003-SD, 2004-SD, 2004-PD, 2005-SD, 2006-SD, 2007-SD, 2008-SD and 2008-PD. This grouping was done so we could investigate: (i) the respective contributions of the successive parental SD and PD cohorts to the genetic pool of this population during the study period, and (ii) a potential link between parents and progeny that were characterized by the same phenotype (SD or PD). In this regard, progeny related to the cohorts produced at a year n and emerging at n+2 (SD) and n+4 (PD), while SD and PD parents related to the cohorts emerging at n but being produced at n–2 and n–4, respectively.

Pairwise Fst were estimated using these 12 groups of individuals to test for genetic differentiation between progeny and SD or PD parents and between SD and PD phenotypes. We also performed assignment tests using all individuals of all cohorts on the basis of their multilocus genotypes using the Bayesian inference method implemented in STRUCTURE 2.3.3 [52]. We used 100,000 burn-in steps followed by 500,000 MCMC simulation steps with a model allowing admixture. The optimal number of genetic clusters (K) represented by the data was determined with the method of Evanno et al. [53], implemented in STRUCTURE HARVESTER [54]. To assess the consistency of results, we performed 25 independent runs for each value of K ranging from 1 to 15 and compared the obtained individual Q-matrices. Results were then graphically displayed using DISTRUCT 1.1 [55].

## Results

### Pattern of Diapause in *M. schimitscheki*

**Figure 2:**
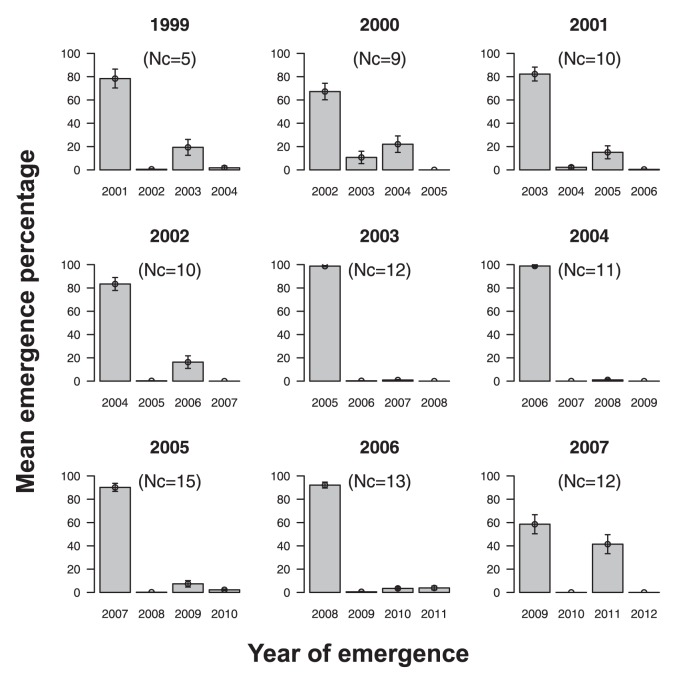
Adult emergences from seeds of *C. atlantica* in southern French cohorts of *M. schimitscheki* over the five consecutive years following their productions (1999–2007). Emergences occurring beyond the 2-year obligatory diapause due to host cone maturation reflect propensities to prolonged diapause. Nc: number of cohorts surveyed per year of production on which mean emergence percentages and their standard errors (bars) were estimated. In 2008, only one population (Mont Ventoux) was surveyed for genetic purposes (Table 2).

Emergences of *M. schimitscheki* spread over a maximum of five years in each cohort, i.e., adult emergences from cohorts produced at a year n (1999–2007) could be recorded at n+2 (2001–2009), n+3 (2002–2010), n+4 (2003–2011) and n+5 (2004–2012), indicating that a fraction of each cohort could have a diapause prolonged by 1, 2 or 3 years (Figure 2). The diapause pattern of *M. schimitscheki* could thus be defined as the respective percentages of individuals emerging at n+2, n+3, n+4 and n+5. In each cohort, we pooled emergence data of male and female wasps as there was no significant effect of sex on the diapause pattern (F = 0.004, df = 1, P = 0.947). As expected, there was a significant effect of diapause duration on emergence proportions (F = 529.45, df = 3, P<0.001). Adult emergences displayed a clear bimodal pattern as wasps mostly emerged after two and four years of diapause (58.5–98.8% and 0.9–41.4%, respectively), while very small fractions of each cohort emerged after three and five years (0.03–10.7% and 0–3.8%, respectively) (Figure 2). Although emergence proportions varied significantly between years of cohort collection and between sites (χ^2^ = 410.84, df = 10, P<0.001 and χ^2^ = 178.75, df = 10, P<0.001, respectively), such a bimodal pattern was conserved among the 97 studied cohorts (Figure 2). To assess whether the bimodal pattern of diapause was specific to *M. schimitscheki* or a common feature in wasp species exploiting true cedars, propensity to prolonged diapause was also estimated in the closely related species *M. pinsapinis*, which lives in sympatry with *M. schimitscheki* in southeastern France and exploits the same ecological niche. *M. pinsapinis* emergences from the same seed lots as *M. schimitscheki* were also spread over a maximum of five years after collection, but conversely to *M. schimitscheki*, *M. pinsapinis* displayed a typical pattern of decreasing emergence frequencies through time (Figure S1). Compared to *M. schimitscheki*, propensity to prolonged diapause was lower in *M. pinsapinis* as 96.3% (±1.1) of individuals emerged after the obligatory 2-year diapause.

### Microsatellite and Population Characteristics

All the cohorts of *M. schimitscheki* sampled at the Mont Ventoux site during the period 1999–2008 displayed the typical bimodal pattern of adult emergence from diapause: the mean emergence percentages after 2-year and 4-year diapauses were estimated at 67.6% (SE = 7.9) and 29.2% (SE = 7.8), respectively (Table 2).

Genotyping all individuals resulted in a matrix of genotypes 98.5% complete (407 out of 413 individuals) for all 9 loci. There was significant LD between loci MS1-110 and MS3-91 in each cohort. The locus MS1-110 was thus removed from the analysis to achieve unbiased and robust results. The HWE tests revealed that none of the ten cohorts x eight loci combinations showed significant departures. All loci were polymorphic in all sampled cohorts (1999–2008) (Table S2). The estimated genetic variability across the eight loci for each cohort is summarized in Table 2 and allelic frequencies per cohort are shown in Table S2. The expected and observed heterozygosities (He and Ho, respectively) from 1999 to 2008 ranged from 0.45 to 0.60 and from 0.55 to 0.69.

### Population Bottlenecks

There was statistical support for population bottlenecks at Mont Ventoux in cohorts produced in 1999, 2001 and 2005 as sign tests under the TPM and SMM hypothesis and the mode shift test were all significant (Table 2). These tests were not proved significant in any other cohorts.

### Temporal Genetic Structure

The genetic differentiation between pooled genetic data from ‘even’ (2000, 2002, 2004, 2006 and 2008) and ‘odd’ (1999, 2001, 2003, 2005 and 2007) cohorts was low and non significant (Fst = 0.2%), suggesting that the bimodal pattern of adult emergence did not influence the temporal genetic structure of the population. The temporal genetic structure of the Mont Ventoux population was also analyzed using pairwise Fst between cohorts. The matrix of pairwise Fst obtained with the correction for the presence of null alleles is given in Table 3. Fst values were significant in 40% of cases (14 out of 36 estimations) but were generally relatively low (1.3–2.9%). The 2004 and the 1999 cohorts were the most frequently significantly differentiated from other cohorts, while the 2005 one was never found to be significantly differentiated (Table 3). These data suggest no clear temporal genetic structure of the Mont Ventoux population of *M. schimitscheki* between 1999 and 2008. In particular, 1999 and 2004 cohorts were as much differentiated from 2-years earlier and 2-years later cohorts (to which they directly relate through SD) as from other cohorts.

**Table 3:** Pairwise Fst divergence between successive cohorts of a natural invasive population of *M. schimitscheki* in southeastern France.

	2001	2002	2003	2004	2005	2006	2007	2008
**1999**	**0.017**	0.014	**0.017**	**0.013**	0.004	0.008	**0.013**	**0.017**
**2001**		−0.002	**0.016**	**0.016**	−0.000	0.008	0.001	0.014
**2002**			0.002	**0.028**	−0.005	0.005	−0.005	−0.004
**2003**				**0.029**	0.004	**0.018**	0.007	0.004
**2004**					0.006	**0.025**	**0.021**	**0.026**
**2005**						0.006	−0.001	−0.001
**2006**							0.013	**0.018**
**2007**								0.004

Sample size for each genotyped cohort is given in Table 2. The 2000 cohort was excluded due to low sample size (N = 5). Pairwise Fst matrix was obtained using all microsatellite loci after applying the correction for null alleles implemented in GENEPOP. Fst values in bold were significantly different from 0 (P<0.05).

The matrix of the pairwise Fst divergences between the 12 groups of individuals of the SD and PD phenotypes is given in Table 4. Fst values were significant in 44% of cases (24 out of 55 estimations) and were generally relatively low (1.2–6.3%). Wasps of both the 2004-SD and 2004-PD groups were the most frequently and the most highly differentiated from the other groups, the 2004-PD wasps displayed the highest levels of genetic differentiation (Table 4). Such greater and more significant Fst values were found between 2004-PD and 2006-SD wasps (6.2%) and between 2004-PD and both 2008-SD and 2008-PD ones (6.3 and 5.6, respectively). Conversely, there was no genetic differentiation between 2006-SD and 2008-SD wasps (P = 0.45), while that found between 2006-SD and 2008-PD was significant but relatively low (Table 4). Allelic frequencies did not reveal any allele that could have been specific to PD phenotypes and that could have further spread in the population (Table S2).

**Table 4:** Pairwise Fst divergence between 12 groups of *M. schimitscheki* produced between 1999 and 2008, expressing either a short (2 years: SD) or a prolonged diapause (4 years: PD) phenotype.

	2001-SD	2002-SD	2003-SD	2004-SD	2004-PD	2005-SD	2006-SD	2007-SD	2008-SD	2008-PD_(26)_
**1999-SD_(32)_**	**0.01693**	0.01372	**0.01713**	0.03948	**0.01199**	0.00368	0.00813	**0.01212**	0.00500	**0.01996**
**2001-SD_(23)_**		−0.00053	**0.01598**	**0.01972**	**0.03520**	−0.00023	0.00773	0.00052	−0.00606	**0.01906**
**2002-SD_(23)_**			0.00276	**0.02336**	**0.05908**	−0.00523	0.00400	−0.00499	−0.01548	−0.00186
**2003-SD_(50)_**				**0.01392**	**0.06935**	0.00446	**0.01776**	0.00721	−0.00717	0.00557
**2004-SD_(50)_**					**0.03867**	0.00471	**0.01429**	**0.01750**	0.00179	**0.02261**
**2004-PD_(38)_**						**0.02912**	**0.06194**	**0.04718**	**0.06339**	**0.05622**
**2005-SD_(50)_**							0.00633	−0.00120	−0.01493	0.00118
**2006-SD_(50)_**								**0.01250**	−0.00373	**0.02256**
**2007-SD_(50)_**									−0.01273	0.00823
**2008-SD_(10)_**										−0.00851

Individuals of the n-SD group were produced at a year n and emerged at year n+2. Individuals of the n-PD group were produced at a year n and emerged at year n+4. Both 2000-PD and 2002-PD groups were excluded from this analysis due to low sample sizes (N<10).This pairwise Fst matrix was obtained using eight microsatellite loci after applying the correction for null alleles implemented in GENEPOP. Fst values in bold were significantly different from 0 (P<0.05). The numbers of genotyped individuals in each group are in subscript.

**Figure 3:**
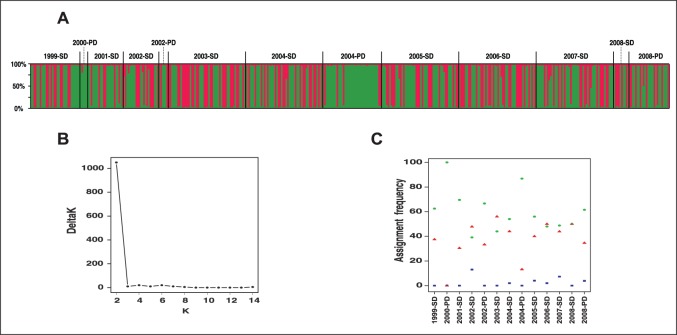
Genetic clustering of ten successive cohorts of *M. schimitscheki* (1999–2008). This Bayesian analysis implemented in STRUCTURE **used** a model allowing admixture and assumed two population clusters (K = 2). A: Graphical representation of the two genetic clusters, where each vertical line represents an individual and each color represents a cluster. Individuals are grouped by year of cohort production and diapause phenotype (SD = 2-year diapause, PD = 4-year prolonged diapause). B: Curve of Evanno's DeltaK corresponding to the STRUCTURE simulations. C: Assignment frequencies of individuals to each cluster in each group (in green and red according to the colors of Figure 3a). Frequencies of admixed individuals are in blue.

We further used Structure on the same data set to infer the temporal genetic structure of the Mont Ventoux population on the basis of individual assignments. Following the method of Evanno et al. [53], ΔK reached a maximum for K = 2, which was thus assumed to reflect the number of genetic clusters in our data set (Figure 3B). A clear structuration pattern was consistently observed in all 25 runs, graphically represented in Figure 3A: two clusters A and B grouped individuals with very high Q-values at the respective frequencies of 56.7 (N = 229, in green) and 40.6% (N = 164, in red) and the frequency of admixed individuals was only 2.7% (N = 11). The frequencies of individuals assigned to clusters A and B were statistically similar in the whole data set (P = 0.4457). There was no clear temporal pattern in this structuration as the frequencies of individuals assigned to each cluster did not significantly increase or decrease during the period of the study (Figure 3C). Similarly, the frequencies of admixed individuals did not increase during the same period (Figure 3C). Clusters A and B were highly differentiated (Fst = 15.6%, P<0.001) and each of them showed significant departures from Hardy-Weinberg equilibrium with a heterozygote deficit (P = 1 and P = 0.99, respectively). Within both 2002 and 2008 cohorts, frequencies of individuals assigned to clusters A and B were similar between SD and PD phenotypes (Fisher exact tests: P = 0.64 and P = 0.47, respectively). Conversely, there was a significant differentiation between SD and PD phenotypes in 2004 as the frequency of individuals assigned to cluster A was significantly higher in 2004-PD wasps than in 2004-SD ones (Fisher exact test, P = 0.002). This indicated that 2004-PD wasps were dominantly assigned to cluster A while 2004-SD ones were assigned to both clusters A and B at similar frequencies.

## Discussion

### An Atypical Pattern of Prolonged Diapause

Following the seminal ecological and evolutionary concepts that were developed for seed germination strategies in desert annual plants [56], prolonged diapause in insects has been well studied in species living in fluctuating environments such as arid, arctic or mountainous habitats [2], [57], [58], or in species specialized on fruits or seeds [33], [59]. In most species with prolonged diapause, the majority of individuals emerge during the first emergence season, which generally reflects the duration of an obligatory diapause, while frequencies of emergences during further years continuously decrease over time [2], [59]. For example, in the chestnut weevil *Curculio elephas,* 61, 35 and 4% of individuals emerge after one, two and three winter diapauses, respectively [14]. In seed-specialized Hymenopterans, all species of the *Megastigmus* genus have a prolonged diapause, which can last up to five years in several cases [24] and display the typical pattern of decreasing frequencies of adult emergence over time [25]. Interestingly, this study presented some contrasts with current literature as it described a particularly atypical pattern of prolonged diapause in insects. Indeed, we showed that adult emergence from diapausing cohorts of *M. schimitscheki* can spread over five years with two consistently major emergence peaks at the second and fourth years, while only a small fraction of a cohort (less than 5% of emerging wasps) emerges at the third and fifth years. Prolonged diapause in *M. schimitscheki* also contrasted with that of *M. pinsapinis*, a closely related sympatric wasp species [35], which also strictly depends on cedar seed resources in France, but for which emergence frequencies decreased constantly over time (Figure S1). This may emphasize the singularity of the diapause strategy of these introduced populations of *M. schimitscheki*.

In seed-specialized wasps feeding on conifers, prolonged diapause is viewed as an adaptive response to spatial and temporal heterogeneity of food supply that concomitantly reduce competition for a limited resource [24], [25]. Indeed, wasps generally have to cope with the masting of their host, which is characterized by interannual quantitative variations in seed production (from mast to null seeding). According to the predator satiation hypothesis, large intermittent seed crops are expected to reduce losses to seed predators by imposing alternations of satiation and starvation periods to predator populations [26]. But extended diapause in insect predators generates more complex interactions and may constrain the predator satiation strategy [60]. The fructification of French *C. atlantica* stands generally follows a 3- or 4-year cycle with two consecutive years of moderate to high seed production and one or two years with an almost null seed production [32], [33]. Although *M. schimitscheki* was found to invest more in prolonged diapause than *M. pinsapinis*, this trait is likely to reflect their adaptation to the *Cedrus* genus [35]. However, if *C. atlantica* is the host of *M. pinsapinis* in both its native (North Africa) and introduction (southern France) areas, one should note that native populations of *M. schimitscheki* in Cyprus did not co-evolve with *C. atlantica* but exclusively with the endemic Cyprus cedar *C. brevifolia* [28]. Little is known about seed production cycles of *C. brevifolia* in Cyprus, but both high population fragmentation levels and adverse environmental conditions are thought to generate longer intervals between mast years than in *C. atlantica*, i.e. four to seven years [61]. In such a context of high unpredictability of the food resource, a bimodal pattern of emergence from diapause could be more advantageous in reducing local extinction risks than a typical unimodal pattern. The diverging strategies of emergence from prolonged diapause observed between French *M. schimitscheki* and *M. pinsapinis* populations could then reflect different host-parasite associations in their respective native areas. Alternatively, as diapause duration is likely to be a multigenic heritable trait [62], differences in emergence strategies could relate to genetic changes resulting directly from the invasion process. Indeed, introduced populations of *M. schimitscheki* were likely founded from an extremely restricted number of individuals and probably had to face substantial interannual variations in food supply in the early stages of their establishment [28]. As a result, new diapause phenotypes could have been shaped by adaptive evolution or neutral changes linked to genetic drift during the establishment process, as seen in many cases of invasion on other traits [63], [64]. But, because the natural history of *M. schimitscheki* in its native area is currently unknown, ascribing a selective interpretation for the diapause pattern observed in France is still questionable. Work is currently in progress to characterize the diapause strategy of native *M. schimitscheki* populations, which may help unravelling the potential effects of phylogeography, demography and/or selection on this trait in France.

Adequate life-history traits may be a key to the establishment and persistence of introduced populations [65]. Prolonged diapause is a major trait for insect population dynamics, especially in fluctuating environments [14], [15]. Recently, low frequency of prolonged diapause (0.1–0.2) has been shown to maximize invasion speed in a stochastic environment by increasing population stochastic growth rate, suggesting that this trait may be advantageous for introduced populations during the colonization phase in such an environment [20]. Our study of 97 different cohorts of *M. schimitscheki* estimated a mean frequency of prolonged diapause of 0.16 (±2.1) between 1999 and 2008. Accordingly to Mahjoub and Menu [20], such a propensity to prolonged diapause could have contributed to population establishment by compensating for demographic and dispersal costs in their new environment.

### Population Bottlenecks

We investigated whether *M. schimitscheki* may have experienced significant reductions in population size at the Mont Ventoux site during the period of the study. Significant population bottlenecks were found only in the cohorts produced in 1999, 2001 and 2005. The use of a rarefaction procedure allowed unbiased estimates of allelic richness and provided the greatest statistical power to detect differences in variation despite a small sample size in some *M. schimitscheki* cohorts. Recent theoretical work suggests that false bottleneck signals may be generated in a population that used to be large and structured [66]. A low genetic diversity can also be the result of a small long-term effective population size instead of a recent population collapse [67]. However, the short history of *M. schimitscheki* in France, its low genetic diversity due a strong founder effect at introduction and the absence of a clear spatial genetic structure since its introduction [28] led us to consider false bottleneck signals unlikely. In species facing significant variations in their resource, effective population size can be severely hampered in years of low resource availability and of the resulting increased competition for this resource. Accordingly, the demography of seed-specialized insects is generally intimately connected to interannual seed abundance levels in their host [24]. Although we lack quantitative information on seed production of *C. atlantica* at Mont Ventoux between 1999 and 2008, the three population bottlenecks detected there may reflect several low seed availability episodes that led to occasional *M. schimitscheki* population collapses. Another possible source of these bottlenecks may relate to the single introduction of *M. schimitscheki* at Mont Ventoux with a severe founder effect [28]. Due to a 2-year obligatory diapause, the bottlenecks detected in the 1999, 2001 and 2005 cohorts may have occurred in the parental generations of 1997, 1999 and 2003 (respectively), which indeed relate to the early history of the wasp in France. Joint effects of initial low genetic diversity, population size, and demographic accidents due to resource fluctuations during the early stages of establishment may then explain the observed bottlenecks. Population bottlenecks generate an erosion of genetic diversity that can lead to genetic differentiation [68], but there was no further evidence of such an impact in the Mont Ventoux population during the period of the study. In this regard, this *M. schimitscheki* population may have progressively reached a sufficiently high effective size to be less susceptible to population collapse, and/or prolonged diapause may act as a genetic refuge promoting temporal gene flow in such a fluctuating environment.

### Prolonged Diapause and Temporal Gene Flow

Focusing on an invasive natural population of *M. schimitscheki* sampled for ten consecutive years at the Mont Ventoux site, we aimed at determining the possible impact of this wasp’s diapause strategy on its temporal genetic structure. We firstly tested whether adult emergences occurring principally after two and four years of diapause may result in significant gene flow disruption, i.e., significant genetic differentiation, between cohorts that were produced in even years (2000, 2002, 2004, 2006 and 2008) and those produced in odd years (1999, 2001, 2003, 2005 and 2007). A non significant Fst value (0.2%) suggested that the consistent bimodal pattern of adult emergence did not disrupt gene flow during this period. Additionally, we did not detect any clear pattern of temporal genetic differentiation between all cohorts using pairwise Fst estimates over the 1999–2008 period (Fst<5%). Three main hypotheses may be formulated to explain these results. First, annual immigration events into the Mont Ventoux population could have prevented local temporal population differentiation [69]. Although we can not formally exclude this hypothesis, historical data however indicate that the spread of *M. schimitscheki* from Mont Ventoux (the likely introduction site) has been biased towards the south-east, mainly due to strong regional prevailing winds travelling towards the south-east (T. Boivin, personal observation) [28]. In this context, the Mont Ventoux population is more likely to act as a source than as a regular sink of migrants in southeastern France. Second, while 3- and 5-year old individuals of an ‘even’ cohort may emerge at low frequencies during an odd year (and vice-versa), their relative contribution to reproduction (i.e., to the genetic pool) the same year may be also modulated by demography. Indeed, even low emergence frequencies may prevent significant genetic drift between ‘even’ and ‘odd’ cohorts [70] and/or generate sufficiently abundant contributors to reproduction when the initial population size is large. Finally, one could expect this population of *M. schimitscheki* to be too recent in France (approximately ten generations) to show any detectable within-population genetic differentiation due to the diapause strategy. In this regard, it will be of critical interest to conduct similar studies within populations with longer histories such as those of the native area in the Middle East (Cyprus, Turkey and Lebanon).

While prolonged diapause has been well described as a demographic refuge against environmental stochasticity [6], [10], [12], its consequence on the structure of neutral population genetics has seldom been investigated. Prolonged diapause is theoretically assumed to promote genetic diversity or an increase of effective population size [17], [71], but empirical tests of these predictions remain scarce [71]. To our knowledge, the role of prolonged diapause in gene flow was exclusively investigated at a spatial scale, in fresh water copepods, for which extended dormancy promoted short-distance dispersal through facilitated transport by vertebrate or wind vectors [71]. For the first time, we have provided here empirical evidence that individuals undergoing prolonged diapause can actively contribute to local temporal gene flow in a natural insect population, i.e., what we call here the role of ‘genetic refuge’ of prolonged diapause.

The STRUCTURE analysis performed on the 1999–2008 cohorts of *M. schimitscheki* at the Mont Ventoux site indicated a strong structuration of this population resulting from two distinct genetic clusters and an extremely low frequency of admixed individuals (Figure 3A). Such a pattern was unexpected, but it was consistent in all 25 runs of our procedure, which suggests that it resulted primarily from a biological phenomenon rather than from a bias in the analysis. This structuration appeared to be temporally stable, rather than transitory, because there was no clear increasing or decreasing trend in the respective frequencies of either cluster A or B, and because admixed individuals remained particularly scarce during the whole period of the study (Figure 3C). The scarcity of admixed individuals also suggested that the conservation of these two distinct genetic clusters did not result from recurrent immigration into the Mont Ventoux population, which would have rather resulted in increasing frequencies of admixed individuals through time. Such a stable structuration pattern, the high inter-cluster differentiation level (Fst = 15.6%) and the significant departures from Hardy-Weinberg equilibrium in each cluster led us to postulate first that we could have dealt with two sub-populations that do not coexist. Our dataset did not allow us to test formally this hypothesis as we could not retrace the precise sampling location of each individual in the field, i.e., clarifying whether all individuals of a given cluster were sampled on trees clearly distinct from those on which all individuals of the other cluster were sampled. Seed-specialized wasps have been shown to select trees in response to diverse visual and olfactory cues [24]. However, the potential for intra-population variation in the responses to these cues and whether there could be significant segregation in the use of space between individuals has never been investigated. A second hypothesis would be that we had dealt with two sub-populations that coexist but that are particularly prone to assortative mating (homogamy), which could support strong genetic divergences in this population without clear spatial isolation. There is now theoretical [72] and experimental evidences for mate choice for close or intermediate relatives in vertebrates [73], but also in insects [74]. Haplodiploidy has been proposed to limit the adverse effects of inbreeding depression as a genetic load that is hidden in heterozygous females should be expressed and purged by selection in the haploid males [75]. But whether the assertions above are likely to apply to this population of *M. schimitscheki* remained enigmatic at this stage of our knowledge of seed wasp’s mating systems.

In the 2004 cohort, the STRUCTURE analysis showed a particularly strong genetic differentiation between SD and PD phenotypes as 2004-SD wasps were equally assigned to both clusters A and B, while 2004-PD wasps were almost exclusively assigned to cluster A (Figure 3A). Such differences in the proportions of both clusters A and B between 2004-SD and 2004-PD wasps were indeed highly significant (P = 0.002), which may partly explain why the highest pairwise Fst values were obtained between 2004-PD and the other groups (Table 4). A similar trend could probably have been observed in the 2002 and 2008 cohorts providing higher sample sizes. But, interestingly, the 2004 data suggested that these two sub-populations A and B (according to the cluster they belong to) strongly diverged in their diapause strategy as follows: sub-population A may express both SD and PD phenotypes in equiprobable proportions, while subpopulation B may principally express SD phenotypes and occasionally both SD and PD phenotypes. In this context, the strong genetic differentiation between both SD and PD phenotypes demonstrated here may support a substantial impact of PD phenotypes to the temporal genetic structure of this *M. schimitscheki* population. Moreover, the atypical bimodal pattern of prolonged diapause and its large interannual variations in propensity suggested that the contribution of PD wasps to reproduction can be quite high in particular years (Table 2).

Although this study could not provide sufficiently strong evidence for this, it may raise critical questions regarding the determinism of prolonged diapause in this wasp and probably other insect species living in fluctuating environments. Indeed, the physiological basis and the factors governing the different stages of the prolonged diapause process (induction, duration, and termination) remain poorly documented. Some studies suggested that the larval prolonged diapause corresponds to physiological processes that are independent of those underlying short (obligatory) diapause and that may be governed by other environmental factors [25]. The chemical composition of seeds during larval development, abiotic factors and genetic and/or non-genetic parental inheritance have been suspected of affecting propensity to prolonged diapause in the seed wasp *M. spermotrophus* [76] and in other cone and seed insects [13]. Further controlled field experiments aiming at testing whether *M. schimitscheki* SD and PD phenotypes produce preferentially SD or PD progeny (respectively) under variable conditions of resource supply could probably help shedding some critical light on these aspects.

### Conclusion

In this study, we described a particularly atypical pattern of prolonged diapause, which is likely to constitute both a demographic and a genetic refuge for invasive forest insects facing interannual fluctuations in resource supply. Propensity to prolonged diapause was estimated from infested seed lots maintained under natural climatic conditions for six consecutive years in rearing boxes, instead of laying on the ground in the forest litter as it occurs in fully natural conditions after cone disarticulation. This procedure favoured an optimal expression of the diapause strategy in the sampled wasp populations, but also allowed diapausing larvae to escape natural post-seed dispersal mortality factors such as pathogens, seed predators or seed decay. This may have led to an overestimation of the frequency of individuals surviving prolonged diapause in field conditions. We were however able to emphasize the genetic influence of PD phenotypes in the field, but further work is needed to assess the survival cost of prolonged diapause [9] in the cedar forest litter. Estimating both optimal and realized prolonged diapause in natural *M. schimitscheki* populations would thus help clarifying the prevalence of PD phenotypes contributing to temporal gene flow.

### Data Accessibility

Microsatellite data: DRYAD entry doi: 10.5061/dryad.c1g08.

## Supporting Information

Figure S1 Adult emergences from seeds of *C. atlantica* in southern French cohorts of *M. pinsapinis* over the five consecutive years following their productions (1999–2007). (DOCX)

Table S1 The primers used to genotype *M. schimitscheki.*
(DOCX)

Table S2 Allele frequencies in the ten consecutive cohorts of *M. schimitscheki.*
(DOCX)
