# Supplementary material for: Temporal Population Genetics of Time Travelling Insects: A Long Term Study in a Seed-Specialized Wasp
Source: PLoS One. 2013 Aug 2;8(8):e70818. doi: 10.1371/journal.pone.0070818 (PMC3732219; doi:10.1371/journal.pone.0070818)

**Figure S1. Adult emergences from seeds of *C. atlantica* in southern French cohorts of *M. pinsapinis* over the five consecutive years following their productions.** Mean emergence percentages and their standard errors (bars) were estimated in 97 cohorts produced in 16 cedar stands between 1999 and 2007. Emergences occurring beyond the 2-year obligatory diapause due to host cone maturation reflect propensities to prolonged diapause.


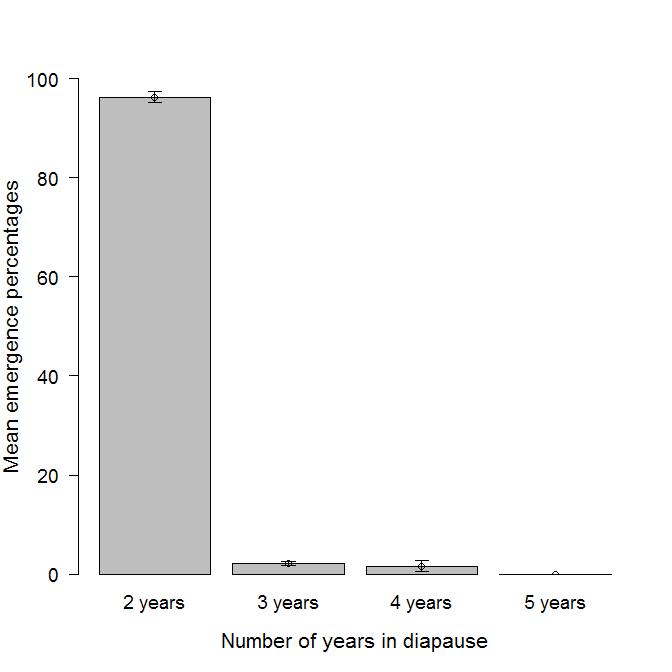

Supplement: Figure S1 — (DOCX) [file pone.0070818.s001.docx]
