# Supplementary material for: Temporal Population Genetics of Time Travelling Insects: A Long Term Study in a Seed-Specialized Wasp
Source: PLoS One. 2013 Aug 2;8(8):e70818. doi: 10.1371/journal.pone.0070818 (PMC3732219; doi:10.1371/journal.pone.0070818)
Supplement: Table S1 — (DOCX) [file pone.0070818.s002.docx]

**Table S1. The primers used to genotype *M. schimitscheki***. The first multiplex used primers MS1-alpha, MS2-162, MW-34, MS1-110, MS3-98 and MS3-105; and the second used MS3-99, MS1-43 and MS3-91.

| **Locus** | **Primer sequence (5’-3’)** | **Forward primer dye** | **Repeat** | **Size range (bp)** | **Genbank accession no** |
| --- | --- | --- | --- | --- | --- |
| MS1-alpha | F : GACTGCAAGCTCGACTCACAC  R : TTTCTCCTCGACGCTGAT | Fam | (CA)18 | 93 – 99 | AY249163.1 |
| MS2-162 | F : GCAGACCGGCGAATAAATAA  R :TCTGCAGCACGATGTAAACG | Fam | (AG)5TG(AG)35 | 122 – 130 | AY249164.1 |
| MW-34 | F : CCCCGCCTCTACCAAATC  R : TTGAAATTGCTCGGACCG | Tamra | (AG)17(G)10 | 144 – 157 | AJ001068.1 |
| MS1-110 | F : TCAGCCCGACTTCGTCCTT  R : AGTCGGCGTTATCGGTTATT | Hex | (AG)51 | 208 – 218 | JX183085 |
| MS3-98 | F:GAGAGAGTCGCGTACGTGTAG  R :TAACGGGTGCTCGAATCAAC | Fam | (GA)28 | 203 – 213 | JX183087 |
| MS3-105 | F : ATGGTCGAGCCCGCTAC  R:GAGGGAGAGACAGACGGCAAAT | Fam | (TC)17(ACTC)5 | 260 – 272 | JX183089 |
| MS3-99 | F : ATTGACTTTGCTCTCCGTCTC  R : CCAAGTTAGCGCTTCAC | Tamra | (CT)52 | 99 – 121 | JX183088 |
| MS1-43 | F : GCAAGCCCTTCGCACAAC  R:GCGCTTCACCGACCTCC | Hex | (AC)15 | 122 – 135 | AY249162.1 |
| MS3-91 | F:CGACACTTATACACCGGCATT  R:CACGTTGCGGACAGAGAGCGA | Tamra | (TC)41 | 211 – 221 | JX183086 |
