# Supplementary material for: Temporal Population Genetics of Time Travelling Insects: A Long Term Study in a Seed-Specialized Wasp
Source: PLoS One. 2013 Aug 2;8(8):e70818. doi: 10.1371/journal.pone.0070818 (PMC3732219; doi:10.1371/journal.pone.0070818)
Supplement: Table S2 — (DOCX) [file pone.0070818.s003.docx]

**Table S2.** **Allele frequencies in the ten consecutive cohorts (1999-2008) of *M. schimitscheki* sampled at Mont Ventoux, France.**

|  |  |  | Cohort (N) | | | | | | | | | |
| --- | --- | --- | --- | --- | --- | --- | --- | --- | --- | --- | --- | --- |
| Locus | Allele | Size | 1999 (32) | 2000 (5) | 2001 (23) | 2002 (29) | 2003 (50) | 2004 (88) | 2005 (50) | 2006 (50) | 2007 (50) | 2008 (36) |
| MS1_alpha |  |  |  |  |  |  |  |  |  |  |  |  |
|  | 1 | 95 | 0.7656 | 0.8000 | 0.7826 | 0.7759 | 0.6300 | 0.7045 | 0.7100 | 0.8300 | 0.7000 | 0.5972 |
|  | 2 | 97 | 0.2344 | 0.2000 | 0.2174 | 0.2241 | 0.3700 | 0.2955 | 0.2900 | 0.1700 | 0.3000 | 0.4028 |
| MS2_162 |  |  |  |  |  |  |  |  |  |  |  |  |
|  | 1 | 113 | 0.0000 | 0.0000 | 0.0000 | 0.0000 | 0.0000 | 0.0057 | 0.0000 | 0.0000 | 0.0000 | 0.0000 |
|  | 2 | 121 | 0.3281 | 0.0000 | 0.2391 | 0.1552 | 0.2700 | 0.2784 | 0.1900 | 0.2900 | 0.2100 | 0.1944 |
|  | 3 | 123 | 0.1875 | 0.2000 | 0.1087 | 0.2069 | 0.2100 | 0.2045 | 0.2100 | 0.2100 | 0.2600 | 0.1667 |
|  | 4 | 125 | 0.0000 | 0.0000 | 0.0000 | 0.0000 | 0.0000 | 0.0000 | 0.0100 | 0.0000 | 0.0100 | 0.0000 |
|  | 5 | 127 | 0.4844 | 0.8000 | 0.6522 | 0.6379 | 0.5200 | 0.5114 | 0.5900 | 0.5000 | 0.5100 | 0.6389 |
|  | 6 | 129 | 0.0000 | 0.0000 | 0.0000 | 0.0000 | 0.0000 | 0.0000 | 0.0000 | 0.0000 | 0.0100 | 0.0000 |
| MS3_105 |  |  |  |  |  |  |  |  |  |  |  |  |
|  | 1 | 258 | 0.1250 | 0.2000 | 0.1522 | 0.2414 | 0.1900 | 0.1761 | 0.2300 | 0.2900 | 0.2200 | 0.2857 |
|  | 2 | 262 | 0.5313 | 0.8000 | 0.4348 | 0.3621 | 0.4100 | 0.5795 | 0.4700 | 0.3300 | 0.4300 | 0.3857 |
|  | 3 | 264 | 0.0000 | 0.0000 | 0.0000 | 0.0000 | 0.0100 | 0.0000 | 0.0000 | 0.0000 | 0.0000 | 0.0000 |
|  | 4 | 268 | 0.0000 | 0.0000 | 0.0000 | 0.0000 | 0.0000 | 0.0057 | 0.0000 | 0.0000 | 0.0100 | 0.0000 |
|  | 5 | 270 | 0.3438 | 0.0000 | 0.4130 | 0.3966 | 0.3900 | 0.2386 | 0.3000 | 0.3800 | 0.3400 | 0.3286 |
| MS1_110 |  |  |  |  |  |  |  |  |  |  |  |  |
|  | 1 | 205 | 0.1094 | 0.1000 | 0.4130 | 0.3621 | 0.1800 | 0.2500 | 0.2900 | 0.2400 | 0.2500 | 0.1389 |
|  | 2 | 207 | 0.0625 | 0.2000 | 0.0000 | 0.0000 | 0.1400 | 0.0852 | 0.0800 | 0.0800 | 0.1100 | 0.1250 |
|  | 3 | 209 | 0.0000 | 0.0000 | 0.0000 | 0.0000 | 0.0000 | 0.0000 | 0.0000 | 0.0000 | 0.0000 | 0.0417 |
|  | 4 | 211 | 0.2656 | 0.1000 | 0.0435 | 0.0345 | 0.1200 | 0.1307 | 0.1100 | 0.1600 | 0.0500 | 0.2222 |
|  | 5 | 213 | 0.4219 | 0.5000 | 0.3478 | 0.4138 | 0.3600 | 0.3807 | 0.3600 | 0.3600 | 0.3800 | 0.3056 |
|  | 6 | 215 | 0.1406 | 0.1000 | 0.1957 | 0.1897 | 0.2000 | 0.1534 | 0.1600 | 0.1600 | 0.2100 | 0.1667 |
| MW_34 |  |  |  |  |  |  |  |  |  |  |  |  |
|  | 1 | 151 | 0.0938 | 0.4000 | 0.1957 | 0.2414 | 0.0800 | 0.1250 | 0.1600 | 0.1000 | 0.3000 | 0.2143 |
|  | 2 | 156 | 0.5313 | 0.6000 | 0.5435 | 0.3793 | 0.3900 | 0.6023 | 0.4800 | 0.4400 | 0.4100 | 0.4143 |
|  | 3 | 157 | 0.3750 | 0.0000 | 0.2609 | 0.3793 | 0.5300 | 0.2727 | 0.3600 | 0.4600 | 0.2900 | 0.3714 |
| MS3_098 |  |  |  |  |  |  |  |  |  |  |  |  |
|  | 1 | 206 | 0.4844 | 0.2000 | 0.6957 | 0.5862 | 0.5900 | 0.4659 | 0.5200 | 0.6000 | 0.6400 | 0.5139 |
|  | 2 | 212 | 0.5156 | 0.8000 | 0.3043 | 0.4138 | 0.4100 | 0.5341 | 0.4800 | 0.4000 | 0.3600 | 0.4861 |
| MS1_43 |  |  |  |  |  |  |  |  |  |  |  |  |
|  | 1 | 117 | 0.0000 | 0.0000 | 0.0000 | 0.0000 | 0.0000 | 0.0057 | 0.0000 | 0.0000 | 0.0000 | 0.0000 |
|  | 2 | 119 | 0.2969 | 0.4000 | 0.3696 | 0.2586 | 0.1600 | 0.2989 | 0.3000 | 0.4200 | 0.2500 | 0.2917 |
|  | 3 | 123 | 0.0000 | 0.0000 | 0.0000 | 0.0000 | 0.0000 | 0.0000 | 0.0000 | 0.0000 | 0.0000 | 0.0139 |
|  | 4 | 125 | 0.1250 | 0.0000 | 0.0217 | 0.0862 | 0.1100 | 0.0747 | 0.0500 | 0.0700 | 0.0600 | 0.0556 |
|  | 5 | 127 | 0.3750 | 0.2000 | 0.3696 | 0.3448 | 0.4200 | 0.3448 | 0.3400 | 0.2800 | 0.3900 | 0.3889 |
|  | 6 | 129 | 0.2031 | 0.4000 | 0.2391 | 0.3103 | 0.3100 | 0.2759 | 0.3100 | 0.2300 | 0.3000 | 0.2500 |
| MS3_91 |  |  |  |  |  |  |  |  |  |  |  |  |
|  | 1 | 211 | 0.0000 | 0.0000 | 0.0000 | 0.0172 | 0.0100 | 0.0632 | 0.0200 | 0.0100 | 0.0100 | 0.0139 |
|  | 2 | 213 | 0.8438 | 0.8000 | 0.6087 | 0.6379 | 0.6000 | 0.6322 | 0.6600 | 0.7500 | 0.6700 | 0.6667 |
|  | 3 | 215 | 0.0000 | 0.0000 | 0.0000 | 0.0172 | 0.0400 | 0.0115 | 0.0000 | 0.0100 | 0.0300 | 0.0278 |
|  | 4 | 217 | 0.1563 | 0.2000 | 0.3913 | 0.3276 | 0.3500 | 0.2874 | 0.3200 | 0.2200 | 0.2900 | 0.2917 |
|  | 5 | 219 | 0.0000 | 0.0000 | 0.0000 | 0.0000 | 0.0000 | 0.0057 | 0.0000 | 0.0100 | 0.0000 | 0.0000 |
| MS3_99 |  |  |  |  |  |  |  |  |  |  |  |  |
|  | 1 | 98 | 0.3125 | 0.5000 | 0.4130 | 0.2931 | 0.3300 | 0.5682 | 0.3800 | 0.4300 | 0.3300 | 0.3000 |
|  | 2 | 100 | 0.3438 | 0.2000 | 0.3043 | 0.4483 | 0.4100 | 0.2102 | 0.3400 | 0.3400 | 0.3500 | 0.5143 |
|  | 3 | 110 | 0.0000 | 0.0000 | 0.0000 | 0.0000 | 0.0000 | 0.0057 | 0.0000 | 0.0000 | 0.0000 | 0.0000 |
|  | 4 | 112 | 0.3438 | 0.3000 | 0.2826 | 0.2586 | 0.2600 | 0.2159 | 0.2800 | 0.2300 | 0.3200 | 0.1857 |
